# Loot box consumption by adolescents pre- and post- pandemic lockdown

**DOI:** 10.7717/peerj.15287

**Published:** 2023-05-01

**Authors:** Whitney DeCamp, Kevin Daly

**Affiliations:** 1 Department of Sociology, Western Michigan University, Kalamazoo, MI, United States of America; 2 Massachusetts Department of Elementary and Secondary Education, Malden, MA, United States of America

**Keywords:** Loot boxes, Video games, Adolescents, Gaming, Gambling

## Abstract

Loot boxes are virtual items that can be redeemed to receive randomly selected other virtual items, and have been criticized for being similar to gambling. The presence of loot boxes in video games has dramatically increased since 2010, with little evidence available for the current prevalence rate of loot box purchasing in the general population, particularly during and following the COVID-19 pandemic lockdowns. This study uses data from representative samples of American youth to estimate prevalence rates for video game play and loot box consumption before the pandemic (2019) and after the pandemic lockdowns (2022) to examine whether the pandemic has affected loot box usage in the general population and among gamers. The results suggest that youth loot box consumption has increased, rising from 24.9% in 2019 to 31.6% in 2022 among 8th grade (age 13–14) youth. The increase over this time period was larger for girls, though boys are still more likely to purchase loot boxes overall.

## Introduction

The COVID-19 pandemic has dramatically reshaped both social and individual behavior. Work closures and remote work, school closures and remote learning, social distancing, and social isolation have all impacted daily routines. As a result of social and individual pandemic-related changes, the average amount of time spent using digital screens has increased, whether for work, school, or entertainment. For some individuals, this increase in screen time includes playing video games, which in turn may increase exposure to loot boxes. Loot boxes have been defined as “virtual items that can be redeemed (opened) to receive randomly selected other virtual items (loot)” (DeCamp, 2021). They have been compared to gambling, with some political and health officials supporting legislation to prohibit youth from buying loot boxes (Macgregor, 2020; Orland, 2019), and are already prohibited under existing gambling restrictions in at least one country (Acres, 2019; Xiao, 2023). Others have argued for the use of warning labels for games that have loot boxes, the use of spending controls or limits, and providing players and parents with better information to make informed decisions (Drummond, Hall & Sauer, 2022). Although research on loot boxes is in its first years and the impacts of engagement are not yet firmly established, the potential increased exposure to loot boxes, particularly among youth, merits further investigation of whether and how the pandemic may have affected consumption. Emerging research suggests that problem gambling, or at least certain forms of it, have increased during the pandemic (Brodeur et al., 2021; Sachdeva, Sharma & Sarangi, 2022), but research on loot boxes, a potential analog to gambling, during the pandemic has been less explored. The present study offers one of the first investigations into prevalence rate changes pre- and post- pandemic lockdowns.

Loot boxes are a fairly recent development in gaming, having been relatively uncommon as purchasable products prior to 2010 (Busby, 2019). The term “loot box” is an umbrella term that is largely interchangeable with similar terms such as loot crate, prize crate, or gacha mechanisms. The digital items may affect gameplay (such as a power-up or a new weapon), may be aesthetic (such as a new costume or skin to customize a character or item), and may be purely for collecting (such as a digital trophy). Depending on the game, loot boxes may be obtained through gameplay by completing objectives, by buying the loot boxes with real-world currency (sometimes with an intermediary game-specific virtual currency), or a combination of the two. The purchasing of loot boxes with real-world currency is asserted to have produced 15 billion US dollars for the video game industry in 2020 (Juniper Research, 2021). The increasing revenue is associated with a substantial proportion of contemporary games containing loot boxes. A review of top mobile games and desktop games found that 58% and 59% of the top games on the Google Play Android store and the Apple iPhone store respectively, and 36% of desktop games contained loot boxes (Zendle et al., 2020). These top games represent over 1.8 billion installs, about half of which were in games rated as suitable for children age seven and older (Zendle et al., 2020). A follow-up study found that the percent of mobile games containing loot boxes had increased to 77% as of mid-2021 (Xiao, Henderson & Newall, 2022).

Purchasing a loot box has many similarities to gambling. Drummond & Sauer (2018) studied 22 popular games, examining them for five characteristics of gambling activities. All 22 of the games they reviewed contained more than one characteristic of a gambling activity, and 45% of the games met all five of the characteristics. Many games have secondary markets where a player can sell virtual items for real-world money as well. A study of three popular games with a secondary market for loot box items found that a total of 1.45 billion sales were made over the course of about two-and-a-half years (Drummond et al., 2020).

Loot box purchasing, much like video game playing itself, varies by age. According to one study, 40% of adult gamers 21 and older have purchased loot boxes (Brooks & Clark, 2019) and 44% of adults have spent money in the past year according to another study (Li, Mills & Nower, 2019). Younger gamers purchase loot boxes at higher rates, with 60% of undergraduates having purchased a loot box (Brooks & Clark, 2019). However, many studies examining prevalence rates have used sampling procedures that may not yield a representative sample. A recent critical review of literature on loot boxes identified this lack of representative samples in loot box research as being a “major problem” in the extant research (McCaffrey, 2023, p. 565), noting that “most loot box studies make use of cross-sectional data from self-selected convenience samples gathered *via* online surveys” (p. 565-566). In their review of empirical studies of loot box engagement, McCaffrey identified only four studies that used representative sampling methods, including two adult samples (von Meduna et al., 2020; Zendle, 2020) and two youth samples (DeCamp, 2021; Kristiansen & Severin, 2020).

von Meduna and colleagues (2020) used a representative sample of German Internet users to study users of Pay2Win games–games that you can pay money to advance—and found that about 39% of players purchase loot boxes, with an average age of 36.7 and being predominately male. The study similarly found that loot box participation and purchasing frequency were associated with problem gambling. Zendle (2020), using quota sampling of adults to form a sample of respondents representative of the United Kingdom’s general population, found that approximately 7.8% of the general population opened loot boxes in the past year. This is similar to responses in the sample for traditional forms of gambling, such as playing bingo in-person (8.2%), games of skill against others (6.8%), and casino table games in-person (7.4%) (Zendle, 2020). Additionally, Zendle (2020) reports a link between loot box purchasing and problem gambling, although smaller than previous studies.

Studies using representative samples found that youth gamers purchase loot boxes at different rates than adult gamers. More specifically, DeCamp (2021), using a representative sample of American youth in Delaware, found that 17% of 11th graders and 25% of 8th graders had purchased loot boxes. The study also found that females were less likely to buy loot boxes and gamble in comparison to males, and that risk and protective factors differed between loot box purchasing and traditional forms of gambling. Kristiansen & Severin (2020), using a representative sample of Danish youth aged 12 to 16 years, found that 42.5% of gamers obtained a loot box (not necessarily buying it) in the past year, 19.8% purchased at least one loot box, and 10.6% sold items in a virtual market. Furthermore, they report large gender differences with 95% of males reporting earning, buying, or selling a loot box compared to 15% of females. Those who bought or sold loot box items made up a higher proportion of at risk or problem gamblers compared to those with no engagement with loot boxes or who just earned them. All of these representative samples were collected prior to the COVID-19 pandemic (von Meduna et al., 2020; Zendle, 2020; DeCamp, 2021; Kristiansen & Severin, 2020). Some studies have been conducted using data collected during the pandemic, although they have not addressed loot box prevelance rates in a general population (*e.g.*, Carey, Delfabbro & King, 2022; Shinkawa et al., 2021). The Gambling Commission (2019, 2022) has produced an annual surveillance reports on youth aged 11-16 in England, Scotland, and Wales, reporting 23% of youth purchased loot boxes in 2019, compared with 24% in 2022. The Gambling Commission did not make direct comparisons or report significance, but the percentages for these years are within each other’s margin of error.

Research on the effects of loot box consumption is in its early stages, but correlational data suggest further investigations are warranted. Among adolescents aged 12-17, loot box purchasing was associated with symptoms of gambling problems, and girls who bought loot boxes viewed gambling more positively than other girls (Rockloff et al., 2021). Further, people who purchase loot boxes are 1.87 times more likely to have psychological distress regardless of demographics on the Kessler-10 psychological distress scale (Drummond, Hall & Sauer, 2022). Moreover, those who bought a loot box are 4.6 times more likely to meet the criteria for gaming disorder. More research is needed to determine whether these effects are causal or co-occurring outcomes.

### COVID-19 pandemic and screen time

The pandemic has dramatically altered social and individual behavior. Business and school closures, as well as quarantine and self-isolation, have had profound effects on the amount of time people spent at home during the pandemic, and potentially moving forward. There has been an increase in depressive symptoms in both boys and girls with the onset and magnitude of the depressive symptoms being more pronounced for girls and being influenced by social connections and loneliness (Liu et al., 2022). To adapt to life during the pandemic, many people altered their regular behavior in a number of ways, including increasing screen time for online work and school, as well as for entertainment. The World Health Organization (2021) even recommended playing social video games as a way of staying connected to others during the pandemic.

Prior to the COVID-19 pandemic, the screen time of children aged 8–12 averaged 4.73 h per day, and for children aged 13–18 the average was 7.37 h per day (Korhonen, 2021). During the pandemic screen time has almost doubled from 11.73 h per week at age 8 before the pandemic, to 23.57 h per week at age 9.5 during the pandemic (McArthur et al., 2021). A meta-analysis of screen time studies during the pandemic supports this, finding an increase of 84 min per day—from 2.7 h per day to 4.1 h per day—across all children and adolescents during the pandemic, with the largest increases being on handheld devices and computers (Madigan et al., 2022). A study of Turkish parents after a long lockdown found that 71.7% of parents said that their child’s screen time had increased to an average of 6.42 h a day (Eyimaya & Irmak, 2021).

Increased screen time has been linked to a number of psychological and physical problems. More physical activity and less screen time are associated with better mental health for children even after controlling for pandemic stressors (Tandon et al., 2021). Increased screen time during the pandemic has also been linked with obesity (Burkart et al., 2022) and the onset of myopia (Wong et al., 2021). At the same time, however, there is support that there are some benefits to playing video games through the pandemic. For example, using screen time as a coping mechanism during the pandemic can promote physical active, educational opportunities, and social support by playing games and online activities to keep social connected (Nagata, Magid & Gabriel, 2020). A study of Italian youth aged 14–19 found that the children benefit from playing video games by having lower health complaints and a higher affective well-being and positive coping (Calandri, Cattelino & Graziano, 2022). However, caution should be taken to limit screen time to mitigate potential negative outcomes, such as poor sleep and excessive sedentary time (Nagata, Magid & Gabriel, 2020).

Increased gaming time has also exposed some to potentially negative effects. A study of rural Chinese adolescent found that increased gaming time was associated with poorer mental health (Li et al., 2022). The study also found that each additional hour of playing video games was associated with increased chance of higher scores on the Depression and Anxiety and Stress Scales (DASS-21). Specific to loot boxes, Hall and colleagues (2021) reported that excessive loot box spending during the pandemic was not more prevalent under isolation conditions, yet the relationship between loot box spending and problem gambling symptomology was more pronounced under isolation. The data used for the study, however, had been collected in the first months of the pandemic, and may not capture more long-lasting effects.

### Current study

Given the concerns over the increased use of entertainment technology during the pandemic, an important related question is whether there were any changes to behavior connected with that technology, such as loot box purchasing. A review of the literature identified no detailed information on loot box purchase prevalence rates before and after the pandemic lockdowns. The present study examines comparable data from before and after the COVID-19 pandemic in order to estimate changes in the consumption of video game loot boxes over this time period. The primary research question is: Has the prevalence rate for loot box purchasing among youth increased during the COVID-19 pandemic? Because prior research has consistently identified a large gender-gap in video game usage (*e.g.*, DeCamp, 2017; Gunter & Daly, 2012), it is necessary to investigate potential gender differences in prevalence rate changes as well. The hypotheses are thus: (1) Prevalence rates for youth video game playing have increased during pandemic lockdowns, (2) Prevalence rates for youth loot box consumption have increased during pandemic lockdowns, and (3) These prevalence rates have increased for both boys and girls.

## Materials & Methods

The data used for this study were collected as part of the Delaware School Survey (DSS), which is an annual surveillance instrument administered by the University of Delaware Center for Drug and Health Studies to adolescents in public and public-charter schools in the State of Delaware. Classrooms for a required subject, such as English, are sampled within participating schools. Because the survey is administered concurrently with other surveys using random classroom selection, any given classroom is eligible only to receive one survey design to avoid participant fatigue. The DSS is presented to students in 5th (age 10-11), 8th (age 13-14), and 11th (age 16-17) grades, though the questionnaire used for 5th grade did not include any questions about loot boxes and the associated data are thus excluded from this study. Participants were asked about loot box consumption in the 2019 and 2022 iterations of the survey. The sample sizes were 2,126 for 2019 8th grade, 3,544 for 2022 8th grade, 2,299 for 2019 11th grade, and 2,948 for 2022 11th grade. The authors of this study do not have the right to share the full third-party data used in this study, although an exact reproduction of the data (created from the frequency tables), limited to the variables used here, is available as a supplemental file to this article. Additionally, interested researchers may request access to the original dataset with other variables by contacting the University of Delaware Center for Drug and Health Studies (2021). This secondary analysis of non-identified data did not require IRB review, although the original data were collected under a protocol approved by the University of Delaware Institutional Review Board and included parental and student informed consent.

Loot box consumption was measured in 2019 using the question: “In the past year, how many loot boxes, loot crates, prize crates, or other packages with random virtual items inside did you buy in video games?” Response options included: “I did not play any video games in the past year”; “I played video games, but did not buy any loot boxes”; “1-5 loot boxes”; “6-10 loot boxes”; “11-20 loot boxes”; and “21 or more loot boxes.” The 2022 instrument contains a few minor wording changes: “During the past 12 months, how many loot boxes, loot crates, prize crates, or other packages containing random virtual items did you buy in video games?” Response options remained the same, except for replacing “year” with “12 months” in the first response option.

In order to measure gender, participants were asked the question: “What is your gender?” Participants in 2022 were given the option to select non-binary (8th *n* = 99; 11th *n* = 73) or to self-describe (8th *n* = 111; 11th *n* = 49), but the number of participants who did so are too few to produce reliable estimates (margin of error = 9% to 14%), so gender-specific analyses are limited to those identifying as boys or girls. For consistency, participants are identified in this article as “boys” or “girls” (rather than males or females) to align with the identities they self-reported in their responses.

Frequencies will be used for each grade and year to establish prevalence rates and are presented in Tables 1 and 2. Chi-square tests will be used to determine statistical significance for differences between years. This procedure will then be repeated for gender-specific subsamples. The two years of data collected for each grade are independent samples with presumably no individual included in both years (it is implausible that someone would be held back three years in a row); all assumptions of the chi-square test have been met. Given that the age difference between the grade samples is three years and that the year samples are three years apart, it is likely that a number of participants in the 2022 11th grade sample were also participants in the 2019 8th grade sample. No comparisons will be made between these two samples (2022 11th grade and 2019 8th grade) given that they are not fully independent samples.

## Results

The prevalence rates for the full samples are presented in Table 3. Among 8th grade participants in 2019, 71.4% reported playing video games in the past year. For 2022, that proportion grew to 77.7%, which is a significant increase (*χ*^2^ = 27.28, *df* = 1, *n* = 5,330, *p* < .001). Likewise, the proportion reporting purchasing loot boxes significant increased from 24.9% to 31.6% (*χ*^2^ = 27.73, *df* = 1, *n* = 5,330, *p* < .001). Among only those playing video games, the proportion who reported purchasing loot boxes significantly increased from 34.9% to 40.7% (*χ* ^2^ = 13.26, *df* = 1, *n* = 4,012, *p* < .001).

**Table 1:** Frequencies (2019).

Response	8th total^a^	8th Boys	8th Girls	11th total^a^	11th Boys	11th Girls
I did not play any video games past 12 months	589	75	510	915	165	744
I played video games, but did not buy any loot boxes	957	504	440	925	600	321
1 to 5 loot boxes	211	174	35	157	128	25
6 to 10 loot boxes	82	74	7	63	60	3
11 to 20 loot boxes	66	61	4	40	33	6
21 or more loot boxes	153	140	12	117	101	16
Total Valid	2058	1028	1008	2217	1087	1115
Missing	68	44	22	82	46	34
Total	2126	1072	1030	2299	1133	1149

**Notes.**

a Total category includes non-binary, self-described gender, and gender non-response, so the number will different from the sum of boys and girls.

**Table 2:** Frequencies (2022).

Response	8th total^a^	8th Boys	8th Girls	11th total^a^	11th Boys	11th Girls
I did not play any video games past 12 months	729	100	601	934	141	760
I played video games, but did not buy any loot boxes	1509	707	684	1210	655	492
1 to 5 loot boxes	414	269	119	249	179	62
6 to 10 loot boxes	215	172	28	92	66	21
11 to 20 loot boxes	147	115	23	64	43	17
21 or more loot boxes	258	208	31	108	87	10
Total Valid	3272	1571	1486	2657	1171	1362
Missing	272	154	106	291	167	114
Total	3544	1725	1592	2948	1338	1476

**Notes.**

a Total category includes non-binary, self-described gender, and gender non-response, so the number will different from the sum of boys and girls.

**Table 3:** Prevalence rates (Full samples).

	2019	2022	+/-	+/- as %	Sig.
8th Grade					
Video game players	71.4%	77.7%	+6.3	+8.9%	^**^
Loot box purchase	24.9%	31.6%	+6.7	+27.0%	^**^
Loot box purchase (Gamers only)	34.9%	40.7%	+5.8	+16.7%	^**^
11th Grade					
Video game players	58.7%	64.8%	+6.1	+10.4%	^**^
Loot box purchase	17.0%	19.3%	+2.3	+13.5%	^*^
Loot box purchase (Gamers only)	29.0%	29.8%	+0.8	+2.8%	
8th Grade (Gender Weighted)					
Video game players	71.6%	77.5%	+6.0	+8.4%	^**^
Loot box purchase	25.0%	31.4%	+6.4	+25.4%	^**^
Loot box purchase (Gamers only)	35.0%	40.5%	+5.5	+15.8%	^**^
11th Grade (Gender Weighted)					
Video game players	58.3%	65.3%	+7.1	+12.1%	^**^
Loot box purchase	16.8%	19.6%	+2.8	+16.5%	^*^
Loot box purchase (Gamers only)	28.8%	29.9%	+1.1	+3.9%	

**Notes.**

** *p* < .01.

* *p* < .05.

Based on 11th grade participants in 2019, 58.7% reported playing video games in the past year. For 2022, that proportion grew to 64.8%, which is a significant increase (*χ*^2^ = 19.22, *df* = 1, *n* = 4,874, *p* < .001). Likewise, the proportion reporting purchasing loot boxes significant increased from 17.0% to 19.3% (*χ*^2^ = 4.29, *df* = 1, *n* = 4,874, p = .038). Among only those playing video games, the proportion who reported purchasing loot boxes changed from 29.0% to 29.8%, which is not a significant increase (*χ*^2^ = 0.24, *df* = 1, *n* = 3,025, p = .625).

In order to control for gender, these analyses were also performed a second time using case weights to control for gender differences by year. Although the change in gender composition between years was small (less than one percent) and non-significant, it is possible for a slight variation in gender to have an impact given the gender disparity in gaming. The results using weighted data to control for gender differences between the 2019 and 2022 samples are presented in the bottom half of Table 3. Although there are some trivial changes to the percentages, the results lead to the same substantive conclusions as from unweighted data.

### Boys

The prevalence rates for boys are presented in Table 4. Among 8th grade participants in 2019 who identified as males, 92.7% reported playing video games in the past year compared to 93.6% of boys in 2022, which is not a significant change (*χ*^2^ = 0.86, *df* = 1, *n* = 2,599, p = .355). However, the proportion reporting purchasing loot boxes significantly increased from 43.7% to 48.6% (*χ*^2^ = 6.13, *df* = 1, *n* = 2,599, p = .013). Among only those playing video games, the proportion who reported purchasing loot boxes significantly increased from 47.1% to 51.9% (*χ*^2^ = 5.38, *df* = 1, *n* = 2,424, p = .020).

**Table 4:** Prevalence rates (Boys).

	2019	2022	+/-	+/- as %	Sig.
8th Grade					
Video game players	92.7%	93.6%	+0.9	+1.0%	
Loot box purchase	43.7%	48.6%	+5.0	+11.3%	^*^
Loot box purchase (Gamers only)	47.1%	51.9%	+4.8	+10.2%	^*^
11th Grade					
Video game players	84.8%	88.0%	+3.1	+3.7%	^*^
Loot box purchase	29.6%	32.0%	+2.4	+8.1%	
Loot box purchase (Gamers only)	34.9%	36.4%	+1.5	+4.2%	

**Notes.**

** *p* < .01.

* *p* < .05.

Looking at 11th grade participants in 2019 who identified as males, 84.8% reported playing video games in the past year compared to 88.0% of boys in 2022, which is a significant change (*χ*^2^ = 4.74, *df* = 1, *n* = 2,258, p =.029). However, the proportion reporting purchasing loot boxes changed from 29.6% to 32.0%, which is not a significant increase (*χ*^2^ = 1.52, *df* = 1, *n* = 2,258, p = .217). Likewise, among only those playing video games, the proportion who reported purchasing loot boxes did not significantly increase, changing only from 34.9% to 36.4% (*χ*^2^ = 0.47, *df* = 1, *n* = 1,952, p = .495).

### Girls

The prevalence rates for girls are presented in Table 5. Among 8th grade participants in 2019 who identified as females, 49.4% reported playing video games in the past year compared to 59.6% of girls in 2022, which is a significant increase (*χ*^2^ = 25.05, *df* = 1, *n* = 2,494, *p* < .001). Likewise, the proportion reporting purchasing loot boxes significantly increased from 5.8% to 13.5% (*χ*^2^ = 38.98, *n* = 2,494, *df* = 1, *p* < .001). Among only those playing video games, the proportion who reported purchasing loot boxes significantly increased from 11.6% to 22.7% (*χ*^2^ = 25.64, *df* = 1, *n* = 1,383, *p* < .001).

**Table 5:** Prevalence rates (Girls).

	2019	2022	+/-	+/- as %	Sig.
8th Grade					
Video Game Players	49.4%	59.6%	+10.2	+20.5%	^**^
Loot Box Purchase	5.8%	13.5%	+7.8	+135.1%	^**^
Loot Box Purchase (Gamers Only)	11.6%	22.7%	+11.1	+95.0%	^**^
11th Grade					
Video Game Players	33.3%	44.2%	+10.9	+32.8%	^**^
Loot Box Purchase	4.5%	8.1%	+3.6	+80.1%	^**^
Loot Box Purchase (Gamers Only)	13.5%	18.3%	+4.8	+35.6%	^*^

**Notes.**

** *p* < .01.

* *p* < .05.

Based on 11th grade participants in 2019 who identified as females, 33.3% reported playing video games in the past year compared to 44.2% of girls in 2022, which is a significant change (*χ*^2^ = 30.69, *df* = 1, *n* = 2,477, *p* < .001). The proportion reporting purchasing loot boxes changed from 4.5% to 8.1%, which is not a significant increase (*χ*^2^ = 13.09, *df* = 1, *n* = 2,477, *p* < .001). Likewise, among only those playing video games, the proportion who reported purchasing loot boxes did not significantly increase, changing only from 13.5% to 18.3% (*χ*^2^ = 3.8416, *df* = 1, *n* = 973, p = .050).

### Gender comparison

Given the stark gender differences in gaming and loot box consumption, supplemental analyses were also performed to directly compare the prevalence rates between boys and girls, and are presented in Table 6. Although the changes from 2019 to 2022 have reduced the gender gap in both gaming and loot box consumption, there still remains a sizable difference in prevalence rates for boys in comparison to girls.

**Table 6:** Prevalence rates (Boys *vs.* Girls).

	Boys	Girls	+/-	+/- as %	Sig.
8th Grade, 2019					
Video game players	92.7%	49.4%	−43.3	−46.7%	^**^
Loot box purchase	43.7%	5.8%	−37.9	−86.8%	^**^
Loot box purchase (Gamers only)	47.1%	11.6%	−35.5	−75.3%	^**^
11th Grade, 2019					
Video game players	84.8%	33.3%	−51.5	−60.8%	^**^
Loot box purchase	29.6%	4.5%	−25.1	−84.9%	^**^
Loot box purchase (Gamers only)	34.9%	13.5%	−21.4	−61.4%	^**^
8th Grade, 2022					
Video game players	93.6%	59.6%	−34.1	−36.4%	^**^
Loot box purchase	48.6%	13.5%	−35.1	−72.2%	^**^
Loot box purchase (Gamers only)	51.9%	22.7%	−29.2	−56.3%	^**^
11th Grade, 2022					
Video game players	88.0%	44.2%	−43.8	−49.7%	^**^
Loot box purchase	32.0%	8.1%	−23.9	−74.8%	^**^
Loot box purchase (Gamers only)	36.4%	18.3%	−18.1	−49.8%	^**^

**Notes.**

** *p* < .01.

* *p* < .05.

## Discussion

Loot box consumption has presumably increased dramatically since 2010 when loot boxes started becoming more available (Busby, 2019), and the effects of engagement with loot boxes is still largely unknown. Although several studies have examined loot box consumption rates, few used representative samples of the population in order to provide generalizable estimates (McCaffrey, 2023). Further, much of the research to date has used data from before the COVID-19 pandemic and thus does not speak to potential increases caused by the increase entertainment screen time associated with the behavioral changes during the pandemic. The present study provided a before-and-after comparison using representative data capture pre- and post- COVID-19 lockdowns to provide an analysis of changing prevalence rates.

The results indicated that adolescents were more likely to play video games after the pandemic than before, supporting the first hypothesis. The results were, however, more mixed for boys, with a statistically significant increase in video game play observed only in 11th grade, resulting in partial support for the hypothesis that these effects will be found for both boys and girls. Youth were also more likely to purchase loot boxes following pandemic lockdowns, supporting the second hypothesis. For the younger adolescents in 8th grade, those who play video games were more likely to purchase loot boxes in 2022 than in 2019, whereas there was no similar increase for older adolescents in 11th grade. Said differently, gamers in 8th grade are more likely to purchase loot boxes after the pandemic, whereas the increase in loot boxes for those in 11th grade is more narrowly driven by the general increase in game playing. Collectively, this suggests that the increased screen time observed during the pandemic (Korhonen, 2021; McArthur et al., 2021) extends to video games and loot box consumption as well. This contradicts the Gambling Commission (2019) and Gambling Commission (2022) reports, which suggest no significant changes over this same time period. Those reports, however, used a United Kingdom sample rather than the present study’s United States sample, so it is possible that the countries have experienced prevalence changes differently. Further, although these data do not make the same kind of comparisons, the changes observed here suggest revisiting early-pandemic results that indicated there were not significant increases in excessive loot box spending resulting from isolation (Hall et al., 2021).

The gender-specific analyses suggest increases in loot box consumption are found among both boys and girls, but are not equally present. For boys in 8th grade, video game playing did not increase, so the increase in loot box purchases were driven by the increased likelihood that gamers would purchase loot boxes. Conversely, boys in 11th grade did not display a significant increase in loot box purchasing in general or among gamers. Girls, however, showed consistent increases in loot box consumption, driven by both increases in video game playing and increased likelihood of purchasing loot boxes among gamers. In both grades, these effects among girls were far larger than their equivalents for boys, with the proportion of girls who purchase loot boxes more than doubling in 8th grade and nearly doubling in 11th grade from 2019 to 2022. This may be related to the gender gap and a ceiling effect on male engagement. Because male youth participation in video games borders on ubiquitous (DeCamp, 2017), the growth potential is more concentrated in female youth. Although the changes varied by gender, however, these findings still identified increases in loot box consumption among both boys and girls. The gender difference in prevalence rates has closed slightly in the three year span analyzed for this study, although boys remain significantly and substantially more likely to play games and to purchase loot boxes.

One limitation of the data used in this study is that they do not include any measurements during the pandemic lockdowns when electronic technology usage presumably would have been most likely to deviate from the normal usage patterns. It is possible, for example, that the usage may have spiked in 2020/2021 and then receded by 2022. Conversely, it is also possible that the 2022 rates reflect a gradual increase since 2019. Further data collection and analysis is needed to determine whether these increases are temporary, will be sustained at current levels, or are part of a continuing trend. Likewise, these data are not able to establish causality with the pandemic, so the increases may be the result of other effects, such as more effective marketing for video games and associated downloadable content, economic changes, or the increased inclusion of loot boxes for purchase in popular games (Xiao, Henderson & Newall, 2022). Additional research is needed to understand the nature of the increased loot box consumption among adolescents, particularly to examine the substantial increases among girls. Regarding age, this study found that adolescents in 11th grade were less likely to play video games and purchase loot boxes than their younger counterparts in 8th grade. Given that loot box purchases require money and, generally, ways to make electronic payments, further investigation into why older youth are less likely to purchase loot boxes would be enlightening.

## Conclusions

The analyses presented here suggest that American youth consumption of loot boxes increased when comparing pre-pandemic rates to post-lockdown rates, with nearly one-third of 8th grade youth reporting buying at least one loot box in the past year. Boys remain more likely to play video games and purchase loot boxes, although the results suggest that the gender gap in these behaviors has narrowed during the pandemic. Purchasing loot boxes has been compared with gambling, although the effects of engagement with loot boxes are as yet not well established. Further research is needed to determine whether loot box engagement has negative outcomes similar to youth gambling, as well as to continue monitoring prevalence rates in this developing market. With lockdowns further in the past and social behavior stabilizing, additional research is needed to determine whether the trends observed here are long-lasting, as well as how changes in availability and marketing may impact loot box consumption patterns over time.

##  Supplemental Information

10.7717/peerj.15287/supp-1 Supplemental Information 1 Reproduction of the data used created from the frequency tables Limited to the variables used here.

## Additional Information and Declarations

### Data Availability

The following information was supplied regarding data availability: The raw data is available in the Supplemental File.
